# Preclinical anti-myeloma activity of EDO-S101, a new bendamustine-derived molecule with added HDACi activity, through potent DNA damage induction and impairment of DNA repair

**DOI:** 10.1186/s13045-017-0495-y

**Published:** 2017-06-20

**Authors:** Ana-Alicia López-Iglesias, Ana B. Herrero, Marta Chesi, Laura San-Segundo, Lorena González-Méndez, Susana Hernández-García, Irena Misiewicz-Krzeminska, Dalia Quwaider, Montserrat Martín-Sánchez, Daniel Primo, Teresa Paíno, P. Leif Bergsagel, Thomas Mehrling, Marcos González-Díaz, Jesús F. San-Miguel, María-Victoria Mateos, Norma C. Gutiérrez, Mercedes Garayoa, Enrique M. Ocio

**Affiliations:** 1 University Hospital of Salamanca (IBSAL) & Cancer Research Center (IBMCC-CSIC-USAL), Salamanca, Spain; 2 Comprehensive Cancer Center, Mayo Clinic, Arizona, USA; 3 Vivia Biotech, Madrid, Spain; 4 Mundipharma-EDO GmbH, Basel, Switzerland; 5 Center for Applied Medical Research (CIMA), IDISNA, University Clinic of Navarra, Pamplona, Spain

**Keywords:** Multiple myeloma, EDO-S101, DNA damage, Homologous recombination, Bendamustine

## Abstract

**Background:**

Despite recent advances in the treatment of multiple myeloma (MM), the prognosis of most patients remains poor, and resistance to traditional and new drugs frequently occurs. EDO-S101 is a novel therapeutic agent conceived as the fusion of a histone deacetylase inhibitor radical to bendamustine, with the aim of potentiating its alkylating activity.

**Methods:**

The efficacy of EDO-S101 was evaluated in vitro*,* ex vivo and in vivo*,* alone, and in combination with standard anti-myeloma agents. The underlying mechanisms of action were also evaluated on MM cell lines, patient samples, and different murine models.

**Results:**

EDO-S101 displayed potent activity in vitro in MM cell lines (IC_50_ 1.6–4.8 μM) and ex vivo in cells isolated from MM patients, which was higher than that of bendamustine and independent of the p53 status and previous melphalan resistance. This activity was confirmed in vivo, in a CB17-SCID murine plasmacytoma model and in de novo Vk*MYC mice, leading to a significant survival improvement in both models. In addition, EDO-S101 was the only drug with single-agent activity in the multidrug resistant Vk12653 murine model. Attending to its mechanism of action, the molecule showed both, a HDACi effect (demonstrated by α-tubulin and histone hyperacetylation) and a DNA-damaging effect (shown by an increase in γH2AX); the latter being again clearly more potent than that of bendamustine. Using a reporter plasmid integrated into the genome of some MM cell lines, we demonstrate that, apart from inducing a potent DNA damage, EDO-S101 specifically inhibited the double strand break repair by the homologous recombination pathway. Moreover, EDO-S101 treatment reduced the recruitment of repair proteins such as RAD51 to DNA-damage sites identified as γH2AX foci. Finally, EDO-S101 preclinically synergized with bortezomib, both in vitro and in vivo.

**Conclusion:**

These findings provide rationale for the clinical investigation of EDO-S101 in MM, either as a single agent or in combination with other anti-MM drugs, particularly proteasome inhibitors.

**Electronic supplementary material:**

The online version of this article (doi:10.1186/s13045-017-0495-y) contains supplementary material, which is available to authorized users.

## Background

Despite recent advances in our understanding of the biology of multiple myeloma (MM) and the development of novel agents and therapeutic strategies [1], the prognosis of MM patients remains poor, and resistance to traditional and new drugs frequently occurs [2]. Alkylators have underpinned the treatment of MM for more than 50 years [3], but short- and long-term toxicity of these drugs remains a concern. Considerable effort has been made to develop new molecules [4] and strategies [5, 6] which could improve the activity and decrease the toxicity of these agents. In this context, EDO-S101 [7] is a first-in-class compound derived from a molecule of the alkylator bendamustine that has been linked to a histone deacetylase inhibitor (HDACi) radical with potent inhibitory activity of both class I and II HDAC [7].

Bendamustine [8] is a bifunctional molecule that combines the alkylating activity of the mustard group with an anti-metabolite purine analog structure. It is approved for newly diagnosed MM patients, based on a randomized phase III study that compared bendamustine and prednisone (BP) with the standard melphalan and prednisone (MP) [9]. However, bendamustine is mainly used in the relapsed setting, either in monotherapy [10] or in combination with bortezomib [5, 6] or immunomodulators [11, 12].

On the other hand, HDACi have been described to inhibit histone deacetylase proteins (HDACs) and other non-histone proteins [13]. HDAC proteins are enzymes that remove acetyl groups from an *N*-acetyl-lysine amino acid on a histone, allowing the histones to wrap the DNA more tightly. HDAC proteins can also be called lysine deacetylases (KDAC), to describe their function rather than their target, which also includes non-histone proteins [14]. HDACs are deregulated in many cancers thereby affecting the expression of tumor suppressors and oncogenes [15]. Two of these HDACi have been tested in phase III trials in combination with bortezomib in relapsed MM patients: vorinostat [16] and panobinostat [17]; particularly, panobinostat potentiated the activity of the proteasome inhibitor, leading to the recent approval of panobinostat in combination with bortezomib and dexamethasone for the treatment of relapsed MM patients.

The rationale for using EDO-S101 arises from the hypothesis that histone acetylation induced by the novel radical would result in a more open chromatin structure which would be particularly susceptible to the alkylating effect of bendamustine. Preclinical data have shown the synergy of panobinostat with melphalan [18] and that of entinostat with bendamustine [19]. Unfortunately, this effect has not been observed in the clinical setting, partly due to the hematological toxicity of these combinations [20, 21]. Our hypothesis is that this hybrid molecule may be able to overcome these caveats.

In the present work, we show the potent activity of EDO-S101 in MM cell lines, in MM cells from patients, in a subcutaneous plasmacytoma xenograft model, and in the clinically predictive VK*MYC murine model. This activity was found to be mediated through the potent induction of DNA damage, deacetylase inhibitory activity, and the simultaneous impairment of DNA damage repair.

## Methods

### Reagents and immunochemicals

EDO-S101 and bendamustine were provided by Mundipharma (Basel, Switzerland). Cell culture media, fetal bovine serum, and penicillin–streptomycin were purchased from Invitrogen Corporation (Gaithersburg, MD, USA). Bortezomib was purchased from LC Laboratories (Woburn, MA, USA), lenalidomide and pomalidomide from Selleckchem (Houston, TX, USA), and dexamethasone and Mirin from Sigma-Aldrich (St Louis, MO, USA). Details of other tested compounds have been specified elsewhere [22].

### MM cell lines, patient samples, and cultures

The origin of the different human MM cell lines and cell cultured methods has been previously reported [23, 24]. Bone marrow (BM) samples from patients with MM were obtained after the approval of the Complejo Asistencial Universitario of Salamanca Review Board and after having obtained informed consent from participating subjects.

### Cell viability

Viability of MM cell lines was examined using the MTT colorimetric assay as previously described [23]. The half-maximal inhibitory concentration (IC_50_) of the drug was calculated using the SigmaPlot software. The cell cycle profile and apoptosis induction were evaluated using commercial kits provided by Immunostep (Salamanca, Spain) as described elsewhere [18].

### Ex vivo analysis of apoptosis in BM samples from myeloma patients

Red blood cells in BM aspirates from patients with MM were lysed, and remaining cellular components were maintained in culture for 48 h in the absence or presence of different concentrations of EDO-S101. EDO-S101 efficacy was measured, ex vivo*,* using an automated flow cytometry platform [25]. For the simultaneous evaluation of the efficacy on plasma cells and toxicity in lymphocytes, a different method was employed [18].

The percentage of cells at each cycle phase was calculated on the alive cells, not considering sub-G0 (apoptotic) cells in the computation.

### Microenvironment assays

MM1S cells were incubated for 48 h with increasing doses of EDO-S101, together with IL-6 at 1 nM or IGF-1 at 10 nM, and proliferation of MM cells was assessed by MTT assay. To evaluate the efficacy of EDO-S101 in the presence of cellular components of the microenvironment, MM1S-luc cells were co-cultured with bone marrow stromal cells (BMSCs) from MM patients. Detailed protocols have been described elsewhere [26].

### Quantification of EDO-S101 synergism with other anti-myeloma agents

MM1S cells were treated for 48 and 72 h with different doses of EDO-S101, and other drugs in monotherapy and in double combinations. The synergism of the combinations was quantitated using the Calcusyn software (Biosoft, Ferguson, MO, USA), which is based on the Chou-Talalay method [27] and calculates a combination index (CI) with the following interpretation: CI > 1: antagonistic effect; CI = 1: additive effect; CI < 1 synergistic effect.

### Immunohistochemistry and immunofluorescence

After appropriate treatments in vitro*,* myeloma cell lines were cytospinned onto glass slides by cytocentrifugation and subjected to immunofluorescence staining as described elsewhere [24]. Immunohistochemical studies were performed on paraffin sections of selected plasmacytomas excised from treated and control mice after two doses of vehicle or EDO-S101 (60 mg/kg) as previously described [26]. Anti-cleaved PARP, anti-phospho-histone H2AX (Ser139), and anti-acetyl-histone H3 were purchased from Cell Signaling, Boston, MA, USA. Anti-Ki67 was obtained from Thermo Scientific, Fremont, CA, USA.

### Western blot

Protein lysates were generated, and western [28] blots performed the following standard procedures [18]. All primary antibodies used in western blot analyses [anti-γH2AX, anti-phospho-CHK1 (Ser345), anti-phospho-CHK2 (Thr68), anti-phospho-BRCA1 (1524), anti-phospho-ATM (Ser 1981), anti-phospho-ATR (Ser 428), anti-p53, anti-acetyl-histone 3, anti-acetyl-histone 4, anti-Bcl-XL, anti-Mcl1, anti-Bcl2 and anti-AIF] were obtained from Cell Signaling, Boston, MA, USA. Horseradish peroxidase linked-donkey (anti-rabbit), sheep (anti-mouse), or mouse (anti-goat) immunoglobulins were used as secondary antibodies at a 1:5000 dilution (Santa Cruz Biotechnology, Santa Cruz, CA, USA).

### Comet assay

Cells were treated with increasing doses of EDO-S101, and collected and processed for alkaline comet assay using a previously described method [28]. Tail moment was calculated at different time points using the OpenComet software [29].

### Homologous recombination functional assay

To determine in vitro levels of homologous recombination (HR), a reporter GFP (green fluorescent protein) plasmid [30] was integrated into the genome of JJN3 and U266 cell lines (JJN3HR and U266HR cells [31]). Then, a unique double strand break (DSB) was introduced by the rare-cutting endonuclease *I-SceI*; a functional GFP gene is then reconstituted by gene conversion, the predominant HR repair pathway in mammalian cells. To evaluate HR efficiency in these cells, they were first pre-incubated with various concentrations of EDO-S101 for 24 h. Then, 1 × 10^6^ cells were co-transfected with 5 μg of an *I-SceI*-expressing plasmid and 0.5 μg of pDsRed-N1 to normalize for transfection efficiency, and incubated again in the presence or absence of EDO-S101 for additional 30 h. Live cells were selected by FSC/SSC gating, and GFP+ and DsRed+ cells were quantified by flow cytometry. HR efficiency was calculated as the ratio of GFP+ to DsRed+ cells.

### Animal models

The two human subcutaneous plasmacytoma models (small and large plasmacytomas) in CB17-SCID mice (The Jackson Laboratory, Bar Harbor, ME, USA) were developed and followed as previously reported [26]. Mice were randomized to the control group (receiving a PBS vehicle solution with 15% HPBCD, 1.5% acetic acid, and 1.25% NaHCO_3_) or the EDO-S101 group, when tumors became palpable (in the case of small plasmacytomas), or when the median tumor volume reached 4000 mm^3^ (for large plasmacytomas). To calculate the working dose of EDO-S101, a maximum tolerated dose (MTD) experiment in CB17-SCID mice was performed. All protocols and experiments were approved by the Animal Ethics Committee of the University of Salamanca.

Two de novo Vk*MYC mice with a M-spike corresponding to gamma/alpha ratio of 0.65 and 0.43 were chosen. EDO-S101 was administered once/week for 2 weeks by intra-cardiac injection at 30 mg/kg in vehicle solution. Bortezomib-resistant tumors generated in the Vk*MYC mice were harvested and passaged serially in mice. Two lines of tumor were generated (Vk12598 and Vk12653) and used for drug testing. For transplantation studies, 7–10-week-old C57BL/6J wt mice were transplanted with ~1 × 10^6^ million splenocytes harvested from Vk12653 tumor-bearing mice. Drug treatment was performed as indicated for the de novo mice and was initiated once their M-spike levels reached >10 g/L, or their gamma/albumin fraction was >0.3 to mimic clinical setting. Details on serum protein electrophoresis (SPEP) analysis as in the generation, characterization, and validation of the Vk*MYC models has been reported elsewhere [22].

### Statistical analyses

Statistical significance of tumor growth inhibition was calculated by Student’s *t* test. Cumulative survival was analyzed using the log rank test. Statistical significance was concluded for values of *p* < 0.05. Analyses were carried out with IBM SPSS Statistics for Windows version 21.0 (IBM Corp., Armonk, NY, USA).

## Results

### EDO-S101 displays more potent anti-myeloma activity than bendamustine, and its activity is independent of previous melphalan resistance and p53 mutational status

**Fig. 1:**
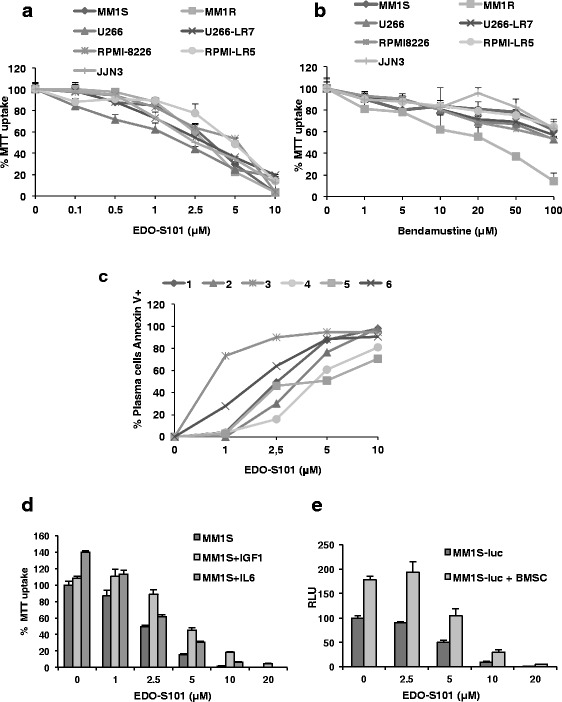
EDO-S101 inhibits the viability of MM cell lines and primary MM cells even in the presence of the microenvironment. Seven MM cell lines were incubated with different concentrations of (**a**) EDO-S101 and (**b**) bendamustine for 48 h. Cell viability was analyzed by MTT reduction. **c** EDO-S101 efficacy was investigated ex vivo on BM samples from six patients with MM. **d** MM1S cells were treated for 48 h with the indicated concentrations of EDO-S101 in the presence or absence of IL-6 (1 nM) or IGF-1 (10 nM). **e** MM1.S-luc cells treated with EDO-S101 in the presence or absence of BMSCs from patients with MM. Cell viability of MM1S-luc cells was analyzed by luminescence, reported in relative light units (RLU), and normalized to that of MM1S-luc cells in monoculture in absence of the drug. Data are expressed as mean ± SD of three independent experiments

The cytotoxic activity of increasing concentrations of EDO-S101 was evaluated on seven MM cell lines with different p53 mutational status and alkylator-resistance profiles. MM1S and MM1R displayed a wild type (WT) p53 and were sensitive to melphalan; RPMI-8226 and U266 and their respective melphalan-resistant counterparts, RPMI-LR5 and U266-LR7, were all p53-mutants. Finally, JJN3 did not express p53, due to a mono-allelic deletion and epigenetic silencing of the other allele [32]. Treatment with increasing doses of EDO-S101 for 48 h reduced cellular viability (Fig. 1a) in all cell lines, with IC_50_ values between 1.6 and 4.8 μM. By contrast, bendamustine showed clearly lower activity with IC_50_ values over 100 μM for all MM cell lines except for MM1R, with an IC_50_ = 20 μM (Fig. 1b). Moreover, EDO-S101 was able to completely overcome the resistance to melphalan in two melphalan-resistant cell lines (U266-LR7 and RPMI-LR5) (Additional file 1: Figure S1).

The effect of EDO-S101 was further investigated ex vivo in cells from the BM from six patients with MM using an automated cytometry method. Four were newly diagnosed, one of them displaying a 1q gain (patient 4), while patients 5 and 6 were relapsing after previous treatments. Patient number 5 had previously received bortezomib, dexamethasone, lenalidomide, and later on an investigational DNA-damaging agent to which he had been refractory; patient number 6, with p53 deletion, had been treated with bortezomib, dexamethasone, and carfilzomib in the context of a clinical trial. EDO-S101 induced cell death in all cases, with a potency similar to that observed in MM cell lines, and without substantial differences between patients despite the different cytogenetic and drug resistance patterns (Fig. 1c). In three additional samples, the activity of EDO-S101 was analyzed not only on plasma cells but also on normal lymphocytes. In all of them (particularly in two), the efficacy on plasma cells exceeded the toxicity on lymphocytes, suggesting a therapeutic window for this compound (Additional file 1: Figure S2).

EDO-S101 was also evaluated in the context of the bone marrow microenvironment. It retained its activity in the presence of the soluble cytokines IL-6 and IGF-1 and when plasma cells were co-cultured with BMSCs from myeloma patients (Fig. 1d, e).

### EDO-S101 activates the DNA damage response and triggers histone and non-histone acetylation

**Fig. 2:**
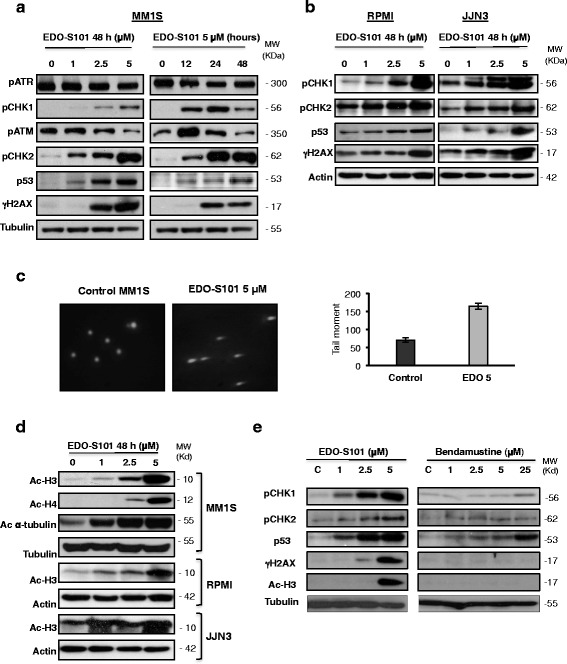
EDO-S101 induces potent DNA damage and histone acetylation. **a** Dose and time response of different proteins implicated in the DNA damage response pathway analyzed on the MM1S cell line. **b** Dose response of different proteins implicated in the DNA damage response pathway on RPMI-8226 and JJN3 cell lines. **c** Comet assay on the MM1S cell line after treatment with EDO-S101 as compared with untreated cells. Images are representatives of at least 20 captures performed in two independent experiments. Mean tail moment was calculated at different time points using the OpenComet software. **d** Dose response (48 h) of acetylated proteins after EDO-S101 treatment in MM1S, RPMI-8226, and JJN3 cell lines. **e** Dose response of proteins implicated in DNA damage repair and acetylation after 48 h of MM1S treatment with EDO-S101 or bendamustine

Treatment of MM1S with EDO-S101 induced an early increase in the DNA damage sensor p-ATM and its downstream effector p-Chk2, which was observed in a dose- and time-dependent fashion. Levels of the other sensor of DNA damage, p-ATR, were not significantly modified by the treatment; however, its downstream effector, p-Chk1, was clearly induced by EDO-S101 (Fig. 2a). Increased levels of p53 and γH2AX, a marker of DNA double strand breaks (DSBs), were observed both in the WT p53 cell line MM1S and also in cells bearing mutated p53, such as RPMI-8226 and JJN3 (Fig. 2b) or U266 (Additional file 1: Figure S3). Consistent with the induction of DNA damage, the comet assay showed characteristic nuclei with tails due to separation of fragmented DNA after treatment with EDO-S101 (Fig. 2c).

Attending to acetylation, 48 h of treatment of MM1S cells with EDO-S101 at doses as low as 1.0 or 2.5 μM markedly increased the acetylation of histones H3 and H4, and of α-tubulin in MM1S (Fig. 2d). Moreover, increased acetylation of histone H3 was also observed in RPMI-8226 and JJN3 cell lines (Fig. 2d).

Finally, the mechanism of EDO-S101 was also evaluated on MM1S cells co-cultured with BMSCs. Interestingly, the EDO-S101-induced DNA damage and HDAC inhibitory effects were maintained even in the presence of stroma components of the bone marrow microenvironment (Additional file 1: Figure S4).

Interestingly, and consistent with the efficacy observed in the MTT assays, all these biological changes were significantly more potently induced by EDO-S101 as compared with bendamustine, even when using this drug at five times higher concentrations (Fig. 2e).

### EDO-S101 induces cell cycle arrest and apoptosis through caspase-independent mechanisms

**Fig. 3:**
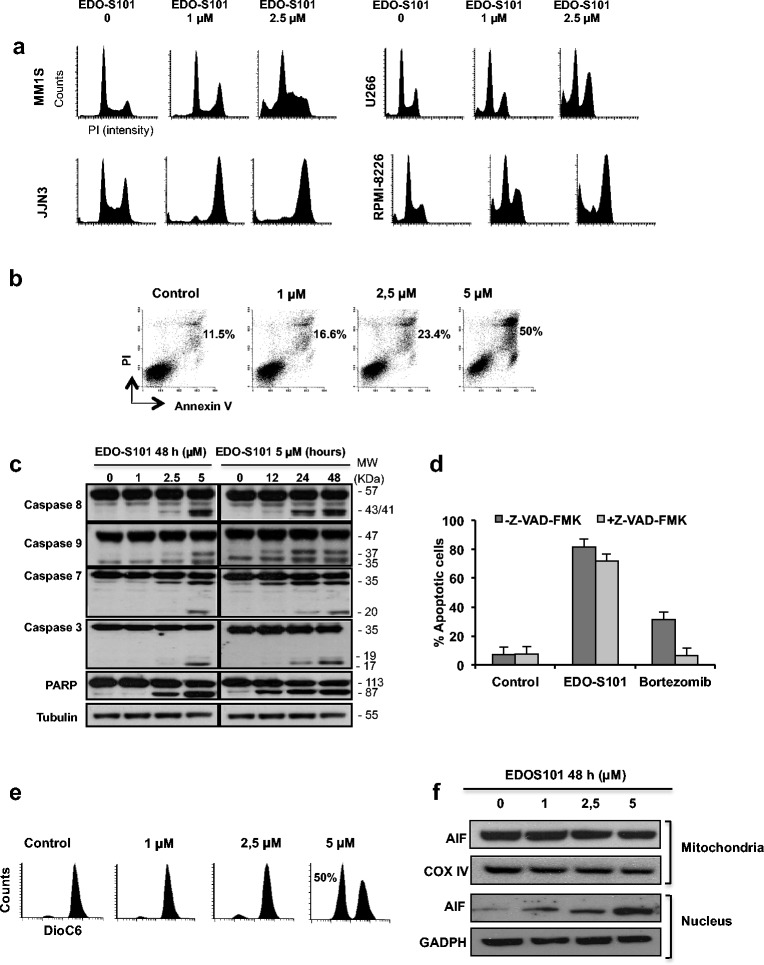
EDO-S101 induces cell cycle arrest, apoptosis, and mitochondrial permeability deregulation. **a** Different MM cell lines were incubated with 1 and 2.5 μM EDO-S101. After propidium iodide (PI) staining, the cell cycle profile was analyzed by flow cytometry. **b** Annexin-V labeling of MM1S cells after treatment with different doses of EDO-S101 for 48 h and evaluated by flow cytometry. **c** Dose and time-response changes of proteins involved in apoptosis after EDO-S101 treatment of MM1S cells. **d** Effect of the pre-incubation for 24 h with the pan-caspase inhibitor Z-VAD-FMK (50 μM) on the apoptosis induced by EDO-S101 at 10 μM. Bortezomib 2 nM was used as a positive control of caspase dependent apoptosis. Data are presented as mean ± SD. **e** Changes in mitochondrial membrane potential after treatment with EDO-S101 as measured by flow cytometry with DioC_6_ staining. **f** Subcellular distribution of AIF in mitochondrial and nuclear fractions, in the MM1S cell line after EDO-S101 treatment

The induction of DNA damage by EDO-S101 prompted the study of potential changes in the cell cycle profile provoked by this agent. Treatment with EDO-S101 caused an accumulation of cells in G2-M phase that was also independent of the p53 mutational status of the cell lines analyzed (Fig. 3a and Additional file 1: Figure S5). The cell cycle arrest was followed by an increase in cell death (sub-G0 population) that was particularly evident at higher doses of EDO-S101 (Fig. 3a).

The induction of apoptosis in MM1S cells after treatment with EDO-S101 was also demonstrated by Annexin-V labeling (Fig. 3b). Western blot analyses showed the dose and time-dependent cleavage of caspases 3, 7, 8, and 9 and of PARP. However, cleavage of this last protein was detected earlier than the activation of the effector caspases, probably suggesting the additional involvement of caspase-independent mechanisms in EDO-S101-induced cell death (Fig. 3c). This was confirmed by only a small reduction in the percentage of apoptotic cells when the MM1S cell line was pre-incubated with the pan-caspase inhibitor, ZVAD-FMK (Fig. 3d).

### EDO-S101 deregulates mitochondrial permeability

Following the activation of the intrinsic apoptotic pathway, there was a decrease in the mitochondrial membrane potential (ΔΨm) with EDO-S101 (Fig. 3e), with the subsequent translocation of AIF from the mitochondria into the nucleus (Fig. 3f). In line with this, a slight decrease of the anti-apoptotic protein Bcl-XL, as well as a translocation of the pro-apoptotic protein Bax to the mitochondria, was observed (Additional file 1: Figure S6); both proteins implicated in ΔΨm decrease, pore formation, and AIF release from the mitochondria [33].

### EDO-S101 inhibits DNA repair by HR in MM

**Fig. 4:**
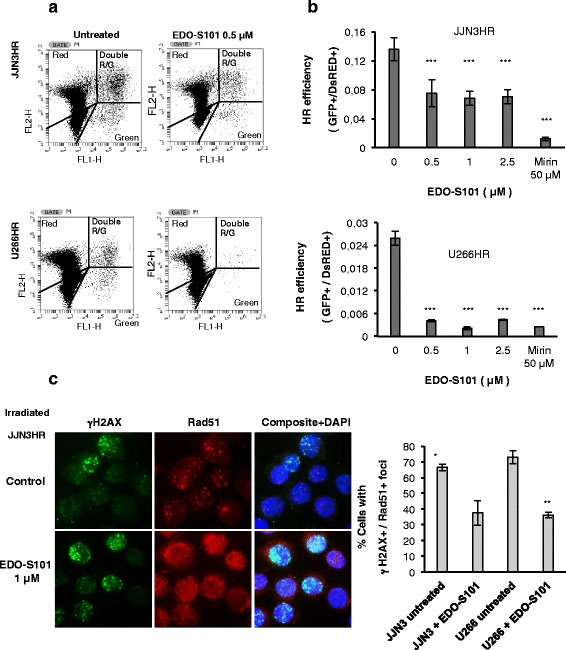
EDO-S101 reduces HR efficiency. **a** JJN3HR and U266HR cells were pretreated with EDO-S101 for 24 h, simultaneously transfected with 5 μg of *I-SceI* endonuclease-expressing plasmid and 0.5 μg of pDSRed2-N1 (*red cells*), and incubated in the presence or absence of EDO-S101 for 30 additional hours at indicated doses. Correct repair by HR of the DSB induced by the endonuclease restored a functional GFP gene whose expression could be detected as *green cells*. **b** Flow cytometry analyses of 100000 GFP+ and/or DsRed+ cells are shown for JJN3HR and of 200000 cells for U266HR. Efficiency of HR is showed on the *right side* and was calculated 30-h post-transfection as the ratio of GFP+ (*green*) to DsRed+ cells (*red*). Data are expressed as the mean of a minimum of three independent experiments ± SD (***p < 0.001, compared to untreated cells). **c** Immunofluorescence assay for γH2AX and RAD51 in JJN3HR cells after 5-h post-irradiation with 2 Gy with or without EDO-S101 treatment. Percentage of foci with double staining for γH2AX and RAD51 are shown on the *right side*. Data are the mean of three independent experiments. One hundred cells were counted in each experiment

Some histone deacetylase inhibitors have been described to inhibit HR in MM [34], and we and others have proposed this mechanism to be important in other tumors such as AML [35], prostate [36] and ovarian [37]. This prompted us to explore the effect of EDO-S101 on HR efficiency in MM cell lines carrying a chromosomally integrated GFP HR reporter cassette (JJN3HR and U266HR). These cells were pretreated with EDO-S101 for 24 h, co-transfected with a *ISceI* endonuclease-expressing plasmid and a pDsRed-N1 plasmid (red) to normalize for transfection efficiency, and incubated again with the drug for 30 additional hours. Mirin, an inhibitor of the Mre11-Rad50-Nbs1 complex required for HR, was used as a control [38]. Correct repair by HR of DSBs induced by the endonuclease restored a functional GFP gene whose expression could be detected by flow cytometry (green cells). There was a significant reduction in the number of HR-proficient cells (green) in both JJN3HR and U266HR cell lines treated with EDO-S101 compared with untreated controls (Fig. 4a). A quantification of the HR efficiency is also shown in Fig. 4b.

Next, we investigated whether EDO-S101 was also involved in the correct recruitment of repair proteins to DNA damage sites. For this purpose, JJN3HR and U266HR cells were pretreated or not with EDO-S101 1 μM for 24 h, and exposed to ionizing radiation (2 Gy) to induce the formation of RAD51^+^ foci. After 5 h, the subcellular localization of RAD51 and γH2AX was analyzed by immunofluorescence. Most JJN3HR control cells (exposed to radiation, but not to EDO-S101 treatment) exhibited discrete RAD51^+^ foci in the nuclei that co-localized with γH2AX**.** However, EDO-S101 partially impaired the recruitment of RAD51 to DNA damage sites expressing γH2AX after radiation, as demonstrated by a more diffuse staining of RAD51, which tended to accumulate in the cytoplasm and a lower percentage of cells exhibiting γH2AX and RAD51 double-stained foci (Fig. 4c). Similar results were obtained in the U266 cell line (not shown).

### EDO-S101 is effective in vivo in a murine plasmacytoma model

**Fig. 5:**
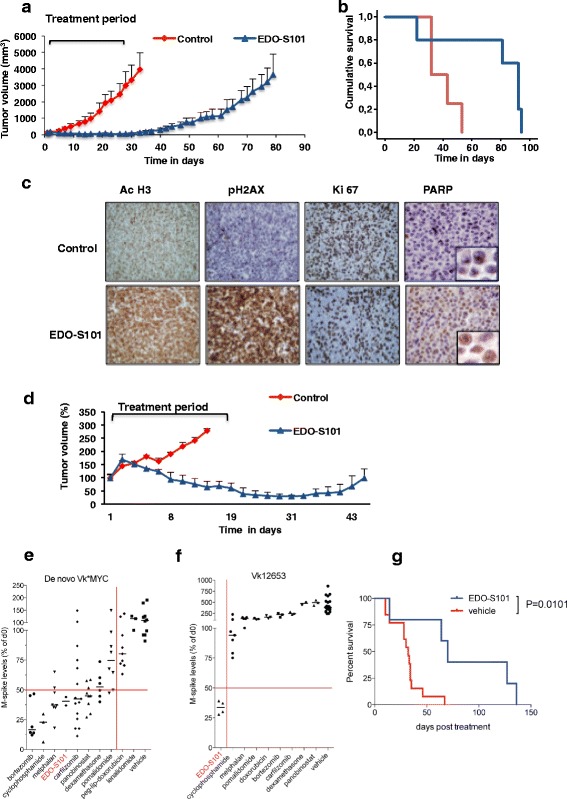
EDO-S101 is active in four xenograft models of human plasmacytoma. **a** CB17-SCID mice (*n* = 5/group) were treated with vehicle (control group) and EDO-S101 (60 mg/kg iv, weekly) for 21 days, and tumor growth evolution was evaluated. **b** Survival of mice in experiment **a** analyzed with a Kaplan–Meier curve. **c** Four CB17-SCID mice (*n* = 2/group) with large plasmacytomas (median of 4000 mm^3^) were administered two consecutive doses of EDO-S101 (60 mg/kg) in two consecutive days, and tumors were then excised to analyze the mechanism of action of EDO-S101. Representative immunohistochemical stainings of big plasmacytomas are shown. **d** Tumor volume evolution of mice with large plasmacytomas (*n* = 2/group), different from experiment **c**, were treated for 21 days at 60 mg/kg. **e** Two de novo Vk*MYC mice with established MM (M-spike >10 g/L) received two weekly doses of 40 mg/kg EDO-S101 by intra-cardiac injection. M-spike levels were measured at day 14 and plotted as percentage of day 0. The response to standard of care agents is shown as a comparison. **f** Four C57BL/6 wild type mice engrafted with Vk12653 MM tumor cells received two weekly doses of 40 mg/kg EDO-S101 by intra-cardiac injection. M-spike levels were measured at day 14 and plotted as percentage of day 0. The response to standard of care agents is shown as a comparison. **g** Kaplan–Meier survival curve of 18 C57BL/6 WT mice transplanted with Vk12653 MM tumor cells and randomized to receive vehicle or two weekly doses of 40 mg/kg EDO-S101 by intra-cardiac injection upon tumor engraftment (M-spike >10 g/L)

The in vivo activity of EDO-S101 was evaluated in several models. In the first one, CB17-SCID mice bearing a xenograft of subcutaneous plasmacytoma of MM1S cells were randomized to receive vehicle or EDO-S101. Treatment with EDO-S101 resulted in strong inhibition of tumor growth that was statistically significant as compared with untreated tumors from day 7 (*p* < 0.05) (Fig. 5a). This translated into a statistically significant advantage in the median survival for EDO-S101 (76 vs 40 days; Log Rank test <0,05; Fig. 5b). Regarding toxicity, mice receiving EDO-S101 lost 10–20% of their body weight; however, all of them spontaneously recovered after the 3 weeks of treatment. There was one mouse that died prematurely on day 23 (tumor growth was continued since this early death did not affect the group median) (Additional file 1: Figure S7).

In a second study, the same groups, times, and doses of treatment were used in mice already bearing large plasmacytomas. Immunohistochemical studies demonstrated a substantial increased staining for histone H3 acetylation and for γH2AX in tumors of mice treated with EDO-S101. Moreover, EDO-S101 showed a decrease in the number of Ki67 positive cells and an increase in PARP positive cells. These findings evidence the potent anti-proliferative and pro-apoptotic effect of this molecule (Fig. 5c shows a representative example of immunostaining with each antibody and condition). Despite the initially large volume of plasmacytomas, EDO-S101 was able to reduce tumor growth (Fig. 5d). Due to ethical reasons, a low number of mice were used in this experiment, which precluded the calculation of Kaplan–Meier curves. Nevertheless, as shown in the figure, EDO-S101 allowed these end-stage mice to remain alive with an acceptable tumor control for more than 40 days.

### EDO-S101 is active against the clinically predictive de novo Vk*MYC and the multidrug refractory Vk12653 models

The Vk*MYC MM mouse model (﻿Additional file 2) has been extensively validated for its clinical predictive value [22, 39, 40]. The single-agent activity of EDO-S101 was evaluated in two aged de novo Vk*MYC mice with established MM, where it induced a significant response (median M-spike 40.5% of day 0), comparable to the most active standard of care anti-MM agents (Fig. 5e). Importantly, such response was sustained for more than 3 months in mice receiving only two doses, 1 week apart. One mouse achieved a complete response 4 weeks after the beginning of the treatment. Remarkably, EDO-S101 was the only drug with single-agent activity in the very aggressive, multidrug resistant Vk12653 transplant model of relapsed/refractory MM (Fig. 5f), where it also significantly prolonged survival (Fig. 5g).

### EDO-S101 potentiates the activity of standard anti-myeloma agents

**Fig. 6:**
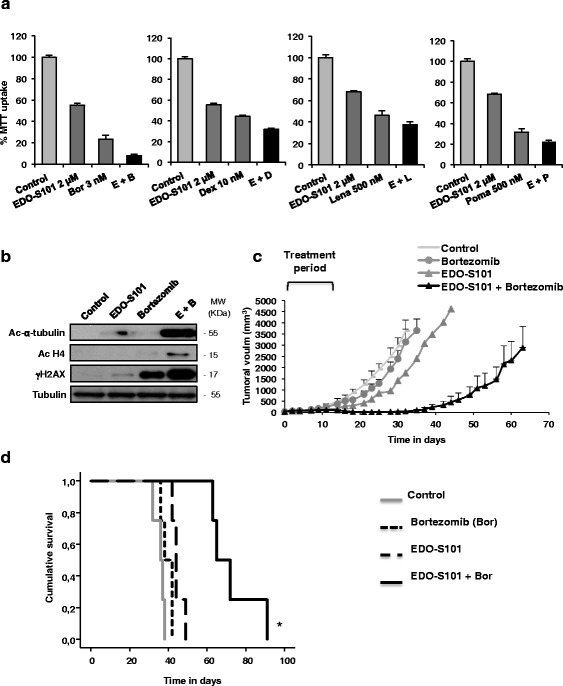
EDO-S101 synergizes with bortezomib in in vitro and in vivo experiments, by potentiating acetylation and DNA damage. **a** MM1S cells were treated with suboptimal concentrations of EDO-S101 and other drugs with anti-myeloma effect for 48 h. **b** Western blot evaluation of the indicated proteins after treatment with bortezomib 3 nM and EDO-S101 2 μM, alone, and in combination for 48 h. **c** Mice bearing a subcutaneous plasmacytoma of MM1S cells were randomized to receive vehicle (control), EDO-S101 (30 mg/kg, iv, weekly), bortezomib (1.25 mg/kg, 2 days per week), and bortezomib + EDO-S101. Differences in tumor growth inhibition were statically significant between the bortezomib + EDO-S101 group and the rest of groups from day 16 (*p* < 0.05). **d** The graphic shows a Kaplan–Meier evaluation of the survival of mice treated as in **c**. (*asterisk* indicates statistical significance. Log Rank *p* < 0.005)

Finally, the ability of EDO-S101 to synergize with conventional anti-MM agents was evaluated. For this purpose, MM1S cells were treated with suboptimal concentrations of EDO-S101 in combination with other anti-MM agents. EDO-S101 showed synergistic CI with all agents in dual combinations: bortezomib (CI 0.6), dexamethasone (CI 0.7), lenalidomide (CI 0.7), and pomalidomide (CI 0.4) (Fig. 6a). The combination with bortezomib was considered particularly promising, since proteasome inhibitors have been the most frequent partners for combination with alkylators in the clinical setting [5, 41]. This effect was confirmed in three additional cell lines: RPMI-8226, JJN3, and U266 (Additional file 1: Figure S8). Regarding the mechanism of action of this combination, the addition of bortezomib enhanced the DNA-damaging effect of EDO-S101, as observed by an increase in γH2AX levels and was also able to potentiate the acetylation induced by EDO-S101 on histone 4 and α-tubulin (Fig. 6b). Moreover, the combination was evaluated in vivo, in mice bearing a subcutaneous plasmacytoma. The combination EDO-S101 plus bortezomib improved the effect of the respective agents in monotherapy (Fig. 6c) and significantly prolonged survival (72 days) as compared to single agent bortezomib and EDO-S101 (39 and 44 days, respectively, Log Rank, *p* < 0.001, Fig. 6d). As far as toxicity is concerned, mice in the EDO-S101 + bortezomib group showed a moderate loss of body weight (always <20%) that spontaneously recovered after the three planned weeks of treatment (Additional file 1: Figure S9).

## Discussion

EDO-S101 represents a new fusion principle in which bendamustine and a deacetylase radical inhibitor have been melted into a new molecule in which functional properties cannot be separated. One hypothesis underlying the design of EDO-S101 is that the combined administration of both in the same compound would increase the efficiency of both mechanisms, while decreasing toxicity and providing more convenient administration. In our studies, EDO-S101 was demonstrated to be more potent than bendamustine, retaining and increasing the alkylating activity with an added deacetylase effect. Moreover, EDO-S101 showed efficacy in all MM cell lines tested (median IC_50_ = 3.1 μM) and fresh plasma cells from untreated and refractory patients (median IC_50_ = 5 μM), independently of the p53 mutational state. It was equally efficient in cell lines resistant to conventional anti-myeloma treatments, such as dexamethasone (MM1R) and melphalan (RPMI-LR5, U266-LR7), indicating that this compound is, at least in vitro, more potent than previous alkylators and could be used to overcome drug resistance. Most importantly, our in vivo data demonstrate that EDO-S101 is also active in in vivo MM models, including the genetically engineered Vk*MYC mouse model, recognized to predict drug response and clinical efficacy in MM. Moreover, it is also remarkable that EDO-S101 is the only drug identified with single-agent activity in the multidrug-resistant Vk12653 transplant model of relapsed/refractory MM (﻿Fig. 5f).

Some previous preclinical studies have shown the synergy of HDACi and alkylators in MM [19, 42] and other B cell lymphoproliferative disorders [43]. However, toxicity has been a concern in the clinical settings. For example, the serious hematological toxicity observed with the combination of panobinostat and melphalan in MM precluded the demonstration of clinical activity in this particular clinical trial [21]. In our ex vivo experiments, we observed a therapeutic window for plasma cells as compared with lymphocytes, which is further confirmed in in vivo studies, in which we showed the clear efficacy of the compound with an acceptable toxicity. This balance was positive, as there was a significant survival benefit in all settings, suggesting that potentially associated secondary effects, such as infections, was not a clear concern for this molecule. However, the safety profile will only be completely defined in the currently ongoing phase I clinical trial in hematological malignancies.

The mechanistic rationale for EDO-S101 was that the HDACi activity would lead to a less compacted structure of the chromatin, making DNA more susceptible to the action of the alkylating molecule. Our in vitro and in vivo studies demonstrated the DNA damage with an increase in H2AX phosphorylation and DNA fragmentation. Attending to the DNA damage pathway and DNA damage response (DDR), EDO-S101 also resulted more active and potent than its precursor, bendamustine, supporting the initial rationale for development.

The pan-HDACi effect of EDO-S101 was also demonstrated by the hyperacetylation of α-tubulin (substrate of the class II deacetylase HDAC6) [44] and histones 3 and 4 (substrates of the class I deacetylases HDAC1 and HDAC2). Moreover, we have also demonstrated that EDO-S101 inhibits DSB repair by HR, which is consistent with previous reports showing a reduction in HR efficiency after treatment with HDACis. In this regard, it has been recently demonstrated the role of HDAC8 in DSB repair, as it co-localizes with RAD51 at DNA damage sites after irradiation, and HDAC8 inhibition resulted in a decrease in RAD51 promoter activity [45]. This could explain the defect in the recruitment of RAD51 to DSBs in irradiated cells pretreated with EDO-S101 as has been shown in this work.

One important consideration for any new agent is whether it synergizes with other standards of care in MM. In this regard, EDO-S101 showed synergy with bortezomib in vitro and in vivo. Several studies have previously reported the preclinical and clinical synergy of alkylating agents plus bortezomib [5, 41] and of bortezomib plus HDACi [17]. Two main mechanisms may explain, at least partially, this synergy: first, the potentiation of the DNA damage induction evidenced by higher γH2AX levels, and second, the simultaneous inhibition of the proteasome induced by bortezomib and the aggresome inhibition exerted by EDO-S101; last, one event evidenced by the increase in α-tubulin acetylation [46]. These circumstances would lead to a great accumulation of unfolded and misfolded proteins [47] which would contribute to cell death.

## Conclusions

In summary, our results demonstrate the in vitro*,* ex vivo, and in vivo anti-myeloma efficacy of EDO-S101 through its HDACi and alkylating activity. The particular mechanism of action of EDO-S101, involving interlaced pathways of potent induction of DNA damage, deacetylase inhibitory activity, and the simultaneous impairment of DNA damage repair, supports the clinical evaluation of this agent in MM patients both in monotherapy and in combination with bortezomib.

## Additional files

Additional file 1: Figure S1. U266, RPMI-8226, and their derivatives, U266-LR7 and RPMI-LR5 partially resistant to melphalan, were incubated with increasing doses of EDO-S101, and cell viability was analyzed by MTT metabolization. Figure S2. EDO-S101 toxicity, on PCs and B lymphocytes derived from bone marrow samples from 3 MM patients, was evaluated after 48 h of incubation by flow cytometry. Figure S3. EDO-S101 dose response (48 h) of different proteins implicated in DNA damage repair in U266 cell line. Figure S4. Dose response (48 h) of different proteins implicated in DNA damage repair and HDAC inhibitory effect after treatment with EDO-S101 of MM1S in the presence or absence of stromal components of the bone marrow microenvironment. MM1S was incubated with EDO-S101 alone, in co-culture with the human stromal cell line hMSC-TERT, and in co-culture with bone marrow mesenchymal stromal cells from a patient with MM (pBMSC). In all cases, the alkylating and the HDACi effect of EDO-S101 were preserved. Figure S5. Different MM cell lines were incubated with 1 and 2.5 μM EDO-S101 for 48 h. After propidium iodide staining, the cell cycle profile was analyzed by flow cytometry. Calculation of percentages of cells at each phase did not consider cells at G0. Figure S6. Bcl-2 family proteins studied by Western blot after treatment of MM1S with the indicated doses of EDO-S101 for 48 h. Figure S7. Toxicity profile of mice bearing a subcutaneus plasmacytoma and treated with the indicated drug. The EDO-S101 group showed a reversible 10–20% loss of body weight. Each point represents the mean ± SD. Figure S8. The combination of EDO-S101 plus bortezomib was also able to improve the effect of single treatments in RPMI-8266, JJN3, and U266 cell lines. Figure S9. Toxicity profile of mice bearing a subcutaneus plasmacytoma and treated with the indicated drugs. The EDO-S101 + Bortezomib group showed a reversible 10–20% loss of body weight. Each point represents the mean ± SD. (PPTX 348 kb)

Additional file 2: Supplemental material and methods. (DOCX 127 kb)

## Abbreviations

AML: Acute myeloid leukemia
BMSCs: Bone marrow stroma cells
CI: Combination index
DDR: DNA damage response
DSB: Double strand break
GFP: Green fluorescent protein
HDACi or DACi: Histone deacetylase inhibitor
HR: Homologous recombination
IC_50_: The half maximal inhibitory concentration
MM: Multiple myeloma
PCs: Plasma cells
PI: Propidium iodide
SD: Standard deviation
WT: Wild type
ΔΨm: Mitochondrial membrane potential
