# Supplementary material for: Preclinical anti-myeloma activity of EDO-S101, a new bendamustine-derived molecule with added HDACi activity, through potent DNA damage induction and impairment of DNA repair
Source: J Hematol Oncol. 2017 Jun 20;10:127. doi: 10.1186/s13045-017-0495-y (PMC5477689; doi:10.1186/s13045-017-0495-y)
Supplement: Supplementary file 1 — U266, RPMI-8226, and their derivatives, U266-LR7 and RPMI-LR5 partially resistant to melphalan, were incubated with increasing doses of EDO-S101, and cell viability was analyzed by MTT metabolization. Figure S2. EDO-S101 toxicity, on PCs and B lymphocytes derived from bone marrow samples from 3 MM patients, was evaluated after 48 h of incubation by flow cytometry. Figure S3. EDO-S101 dose response (48 h) of different proteins implicated in DNA damage repair in U266 cell line. Figure S4. Dose response (48 h) of different proteins implicated in DNA damage repair and HDAC inhibitory effect after treatment with EDO-S101 of MM1S in the presence or absence of stromal components of the bone marrow microenvironment. MM1S was incubated with EDO-S101 alone, in co-culture with the human stromal cell line hMSC-TERT, and in co-culture with bone marrow mesenchymal stromal cells from a patient with MM (pBMSC). In all cases, the alkylating and the HDACi effect of EDO-S101 were preserved. Figure S5. Different MM cell lines were incubated with 1 and 2.5 μM EDO-S101 for 48 h. After propidium iodide staining, the cell cycle profile was analyzed by flow cytometry. Calculation of percentages of cells at each phase did not consider cells at G0. Figure S6. Bcl-2 family proteins studied by Western blot after treatment of MM1S with the indicated doses of EDO-S101 for 48 h. Figure S7. Toxicity profile of mice bearing a subcutaneus plasmacytoma and treated with the indicated drug. The EDO-S101 group showed a reversible 10–20% loss of body weight. Each point represents the mean ± SD. Figure S8. The combination of EDO-S101 plus bortezomib was also able to improve the effect of single treatments in RPMI-8266, JJN3, and U266 cell lines. Figure S9. Toxicity profile of mice bearing a subcutaneus plasmacytoma and treated with the indicated drugs. The EDO-S101 + Bortezomib group showed a reversible 10–20% loss of body weight. Each point represents the mean ± SD. (PPTX 348 kb) [file 13045_2017_495_MOESM1_ESM.pptx]

## Slide 1
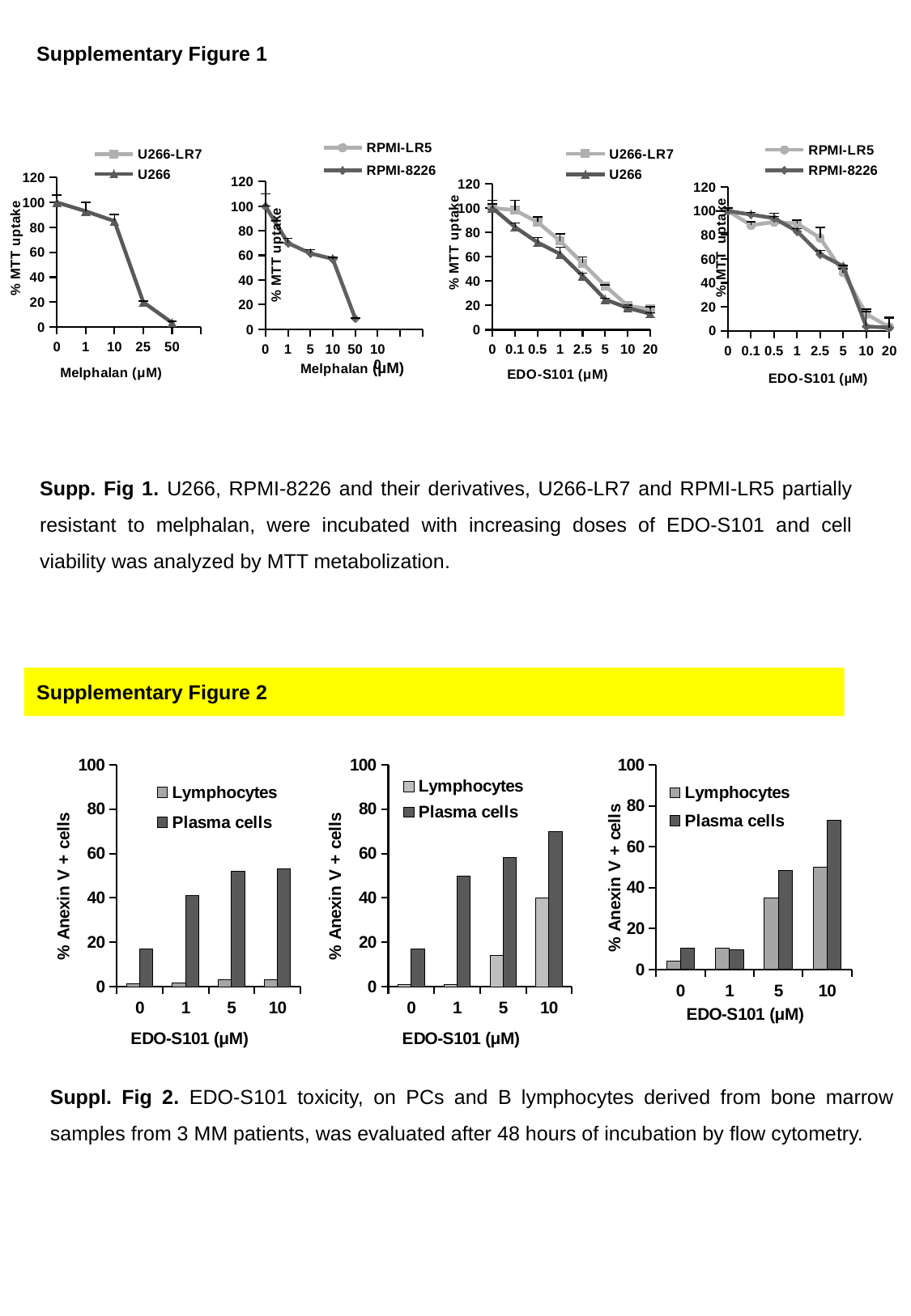

# Supplementary Figure 1
### Chart
| Category | | |
|---|---|---|
| 0.0 | 100.0 | 100.0 |
| 1.0 | 93.11275643991978 | 92.95487864473041 |
| 10.0 | 93.57550516736045 | 85.0301352011728 |
| 25.0 | 76.56177695511337 | 20.02769180648315 |
| 50.0 | 67.07542804256823 | 3.485909757289461 |
### Chart
| Category | | |
|---|---|---|
| 0 | 100.0 | 100.0 |
| 0.1 | 88.11298548450371 | 96.96644593244795 |
| 0.5 | 90.80685236040205 | 93.99948207613355 |
| 1 | 89.49588073754305 | 83.02689504642593 |
| 2.5 | 77.49880127283028 | 63.95989789500909 |
| 5 | 48.66287432980254 | 53.81968850578804 |
| 10 | 13.9466457434288 | 3.605736623383028 |
| 20 | 3.151562704328493 | 2.569888892999393 |
[unsupported chart]
### Chart
| Category | | |
|---|---|---|
| 0 | 100.0 | 100.0 |
| 0.1 | 98.1345002303086 | 84.30879129799253 |
| 0.5 | 88.2875275090844 | 71.69413101712735 |
| 1 | 72.95409181636728 | 62.18848932543224 |
| 2.5 | 54.6368800859819 | 44.18378115106599 |
| 5 | 36.02026715799119 | 24.84779608734475 |
| 10 | 19.63508879676544 | 17.90188597559326 |
| 20 | 16.96606786427146 | 13.0773601753389 |
Supp. Fig 1. U266, RPMI-8226 and their derivatives, U266-LR7 and RPMI-LR5 partially resistant to melphalan, were incubated with increasing doses of EDO-S101 and cell viability was analyzed by MTT metabolization.
Supplementary Figure 2
### Chart
| Category | | |
|---|---|---|
| 0.0 | 1.2 | 17.0 |
| 1.0 | 1.6 | 41.0 |
| 5.0 | 3.0 | 52.0 |
| 10.0 | 3.0 | 53.0 |
### Chart
| Category | | |
|---|---|---|
| 0.0 | 1.0 | 17.0 |
| 1.0 | 1.0 | 50.0 |
| 5.0 | 14.0 | 58.2 |
| 10.0 | 40.0 | 70.0 |
### Chart
| Category | | |
|---|---|---|
| 0.0 | 4.22 | 10.6 |
| 1.0 | 10.4 | 9.8 |
| 5.0 | 35.0 | 48.51 |
| 10.0 | 50.0 | 73.0 |Suppl. Fig 2. EDO-S101 toxicity, on PCs and B lymphocytes derived from bone marrow samples from 3 MM patients, was evaluated after 48 hours of incubation by flow cytometry.

## Slide 2
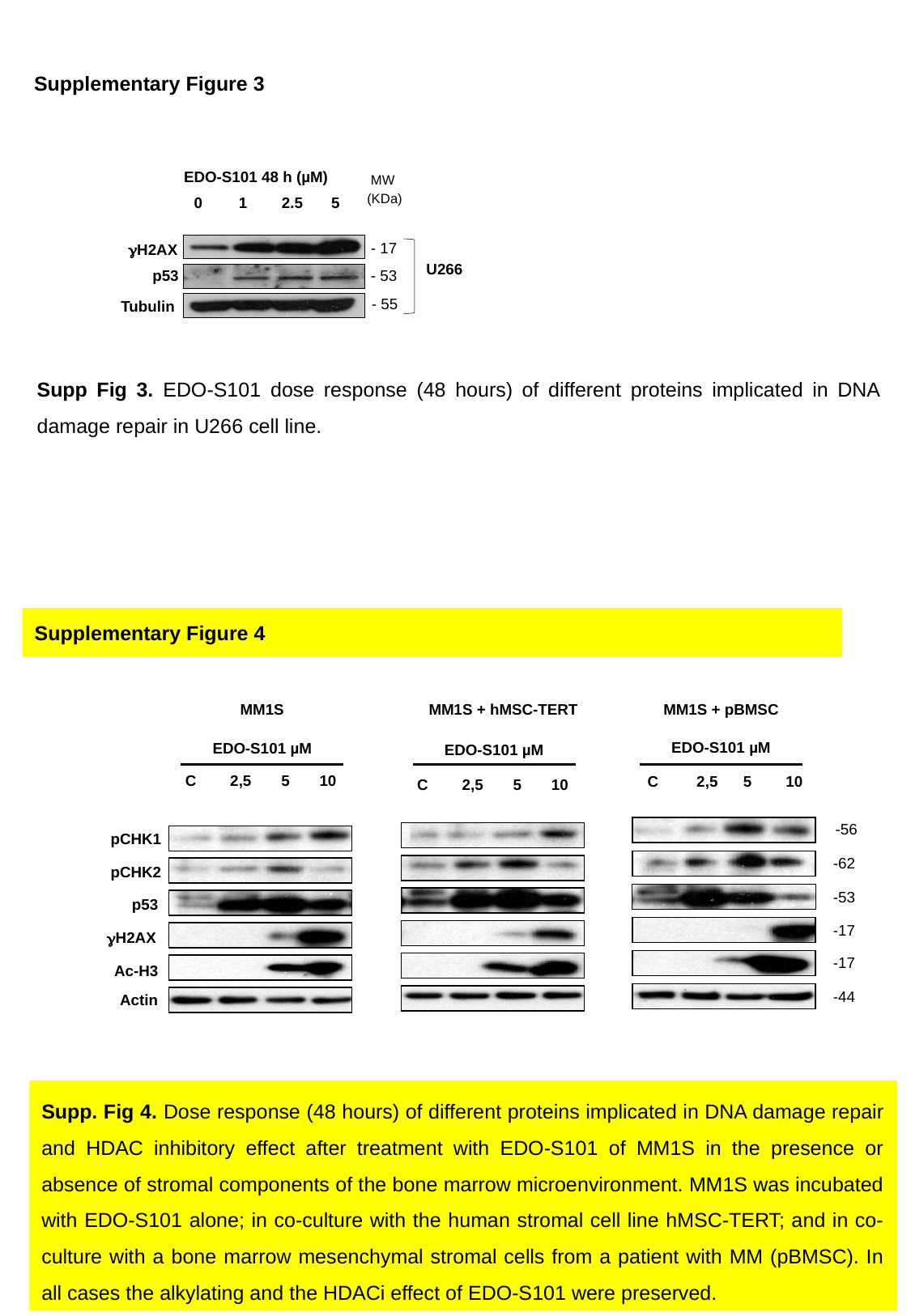

Supplementary Figure 3
EDO-S101 48 h (µM)
MW
 (KDa)
0
1
 2.5
 5
- 17
γH2AX
U266
 p53
- 53
- 55
Tubulin
Supp Fig 3. EDO-S101 dose response (48 hours) of different proteins implicated in DNA damage repair in U266 cell line.
Supplementary Figure 4
MM1S
MM1S + hMSC-TERT
MM1S + pBMSC
EDO-S101 µM
EDO-S101 µM
EDO-S101 µM
 C 2,5 5 10
 C 2,5 5 10
 C 2,5 5 10
 -56
pCHK1
-62
pCHK2
-53
p53
-17
γH2AX
-17
Ac-H3
-44
Actin
Supp. Fig 4. Dose response (48 hours) of different proteins implicated in DNA damage repair and HDAC inhibitory effect after treatment with EDO-S101 of MM1S in the presence or absence of stromal components of the bone marrow microenvironment. MM1S was incubated with EDO-S101 alone; in co-culture with the human stromal cell line hMSC-TERT; and in co-culture with a bone marrow mesenchymal stromal cells from a patient with MM (pBMSC). In all cases the alkylating and the HDACi effect of EDO-S101 were preserved.

## Slide 3
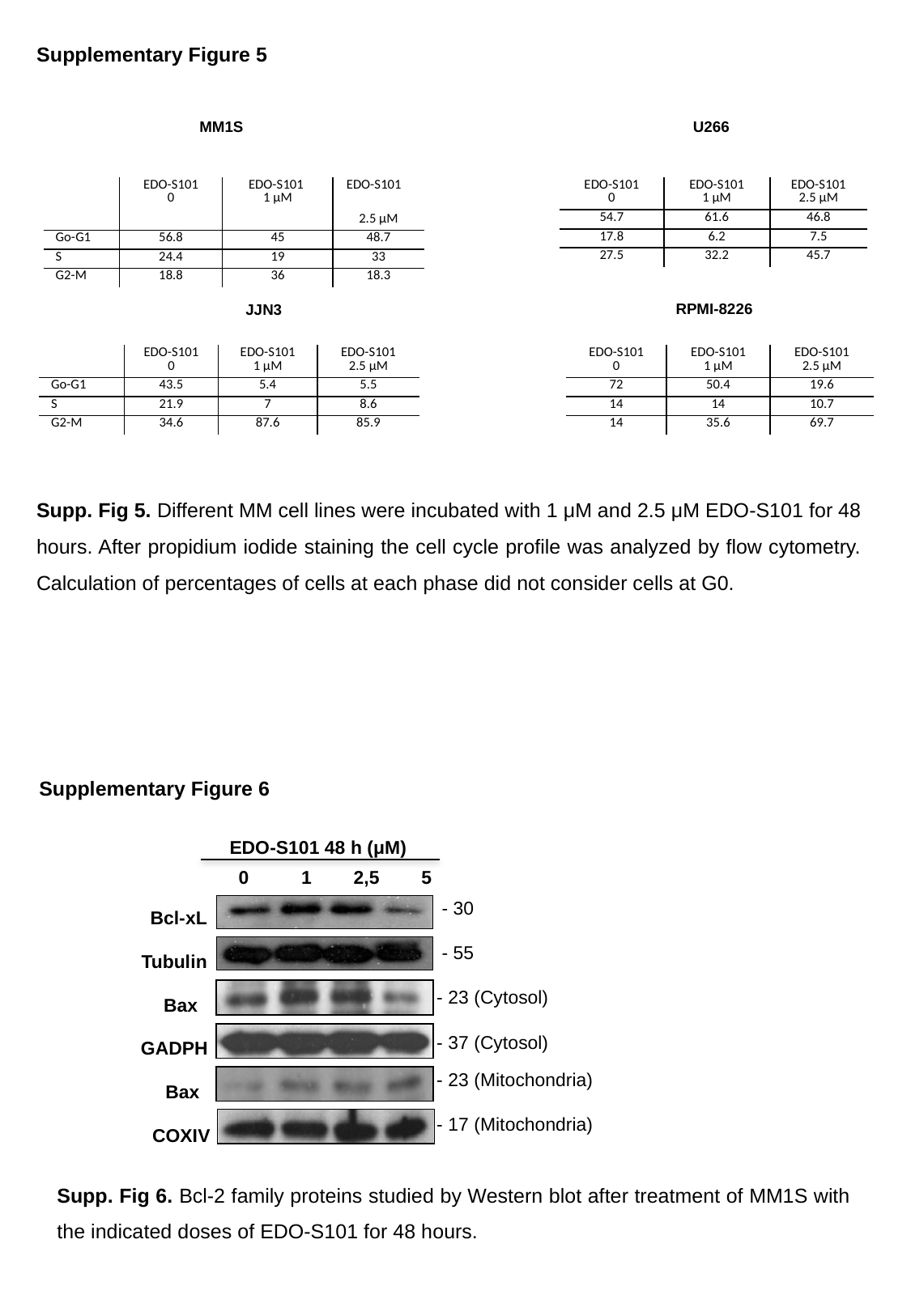

Supplementary Figure 5
MM1S
U266
| | EDO-S101 0 | EDO-S101 1 μM | EDO-S101 2.5 μM |
| --- | --- | --- | --- |
| Go-G1 | 56.8 | 45 | 48.7 |
| S | 24.4 | 19 | 33 |
| G2-M | 18.8 | 36 | 18.3 |
| EDO-S101 0 | EDO-S101 1 μM | EDO-S101 2.5 μM |
| --- | --- | --- |
| 54.7 | 61.6 | 46.8 |
| 17.8 | 6.2 | 7.5 |
| 27.5 | 32.2 | 45.7 |
RPMI-8226
JJN3
| | EDO-S101 0 | EDO-S101 1 μM | EDO-S101 2.5 μM |
| --- | --- | --- | --- |
| Go-G1 | 43.5 | 5.4 | 5.5 |
| S | 21.9 | 7 | 8.6 |
| G2-M | 34.6 | 87.6 | 85.9 |
| EDO-S101 0 | EDO-S101 1 μM | EDO-S101 2.5 μM |
| --- | --- | --- |
| 72 | 50.4 | 19.6 |
| 14 | 14 | 10.7 |
| 14 | 35.6 | 69.7 |
Supp. Fig 5. Different MM cell lines were incubated with 1 μM and 2.5 μM EDO-S101 for 48 hours. After propidium iodide staining the cell cycle profile was analyzed by flow cytometry. Calculation of percentages of cells at each phase did not consider cells at G0.
Supplementary Figure 6
EDO-S101 48 h (μM)
 0 1 2,5 5
 - 30
Bcl-xL
 - 55
Tubulin
- 23 (Cytosol)
Bax
- 37 (Cytosol)
GADPH
- 23 (Mitochondria)
Bax
- 17 (Mitochondria)
COXIV
Supp. Fig 6. Bcl-2 family proteins studied by Western blot after treatment of MM1S with the indicated doses of EDO-S101 for 48 hours.

## Slide 4
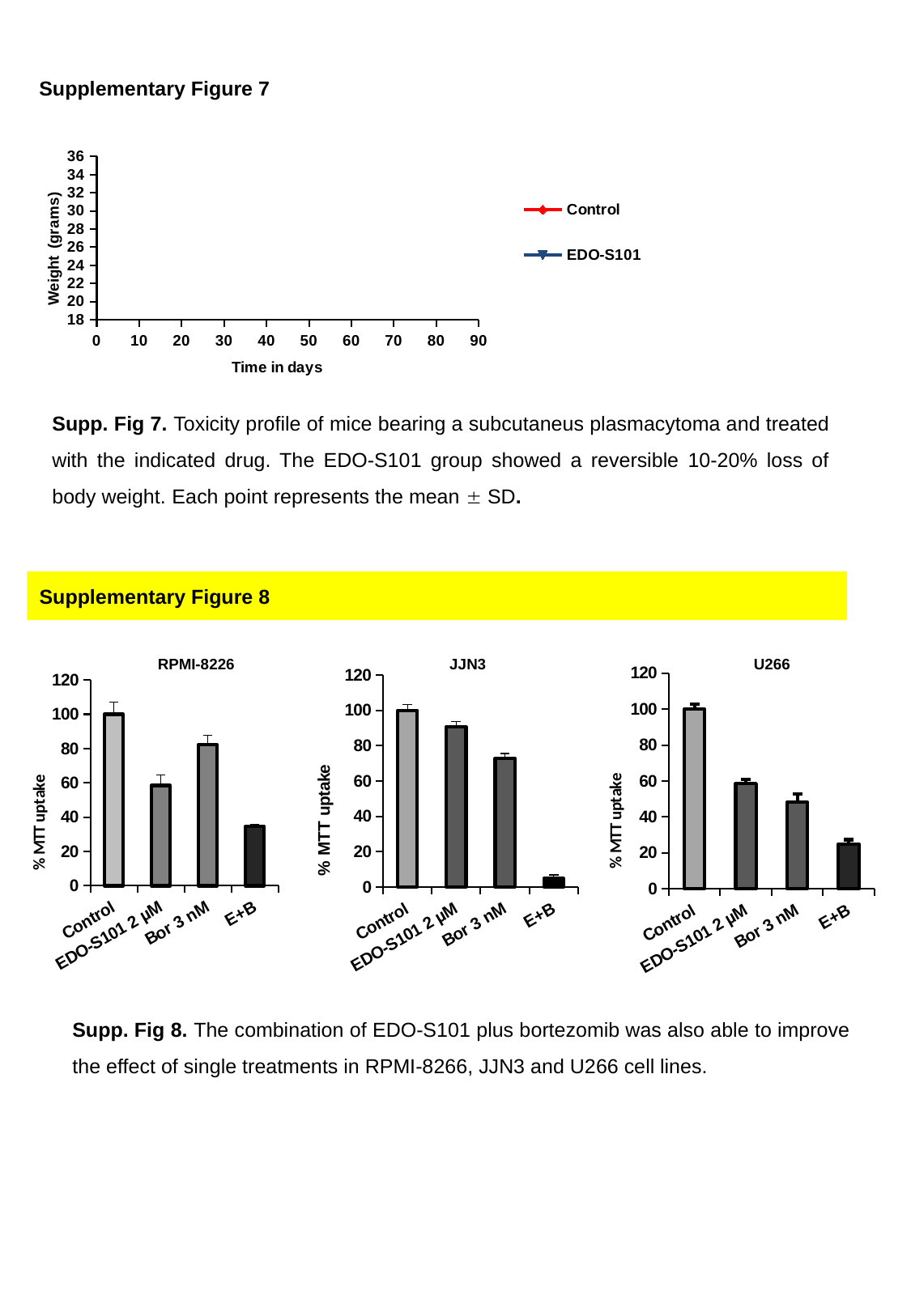

# Supplementary Figure 7
### Chart
| Category | Control | EDO-S101 |
|---|---|---|Supp. Fig 7. Toxicity profile of mice bearing a subcutaneus plasmacytoma and treated with the indicated drug. The EDO-S101 group showed a reversible 10-20% loss of body weight. Each point represents the mean ± SD.
Supplementary Figure 8
### Chart
| Category | |
|---|---|
| Control | 100.0 |
| EDO-S101 2 µM | 58.45287815507535 |
| Bor 3 nM | 82.25894316324677 |
| E+B | 34.58325767205375 |
### Chart
| Category | |
|---|---|
| Control | 100.0 |
| EDO-S101 2 µM | 58.57707509881423 |
| Bor 3 nM | 48.16385195831837 |
| E+B | 24.86525332375134 |
### Chart
| Category | |
|---|---|
| Control | 100.0 |
| EDO-S101 2 µM | 90.82928015564201 |
| Bor 3 nM | 72.94503891050573 |
| E+B | 4.83463035019455 |RPMI-8226
JJN3
U266
Supp. Fig 8. The combination of EDO-S101 plus bortezomib was also able to improve the effect of single treatments in RPMI-8266, JJN3 and U266 cell lines.

## Slide 5
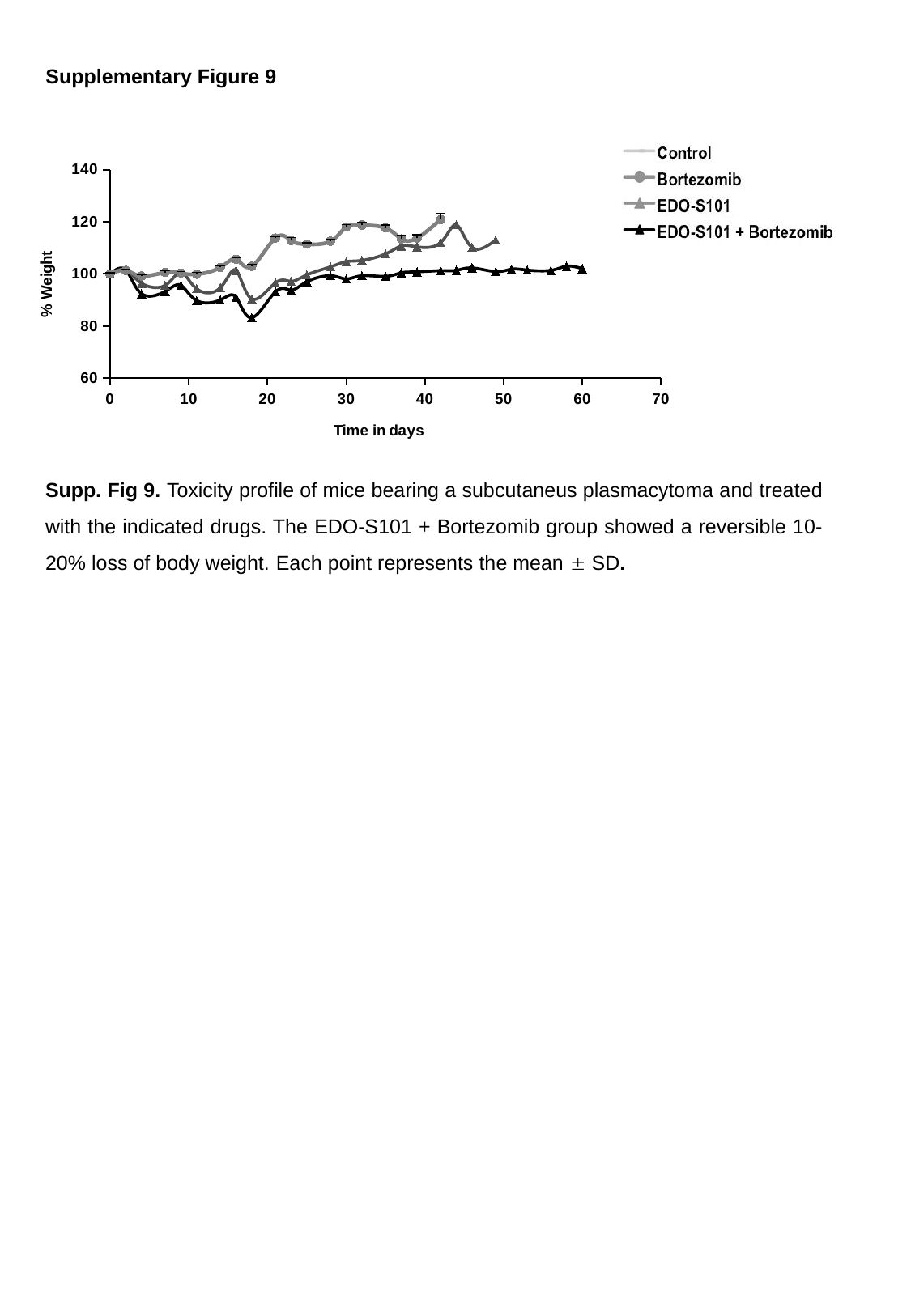

Supplementary Figure 9
### Chart
| Category | Control | Bor | EDO | BorEDO |
|---|---|---|---|---|Supp. Fig 9. Toxicity profile of mice bearing a subcutaneus plasmacytoma and treated with the indicated drugs. The EDO-S101 + Bortezomib group showed a reversible 10-20% loss of body weight. Each point represents the mean ± SD.
