# Supplementary material for: Preclinical anti-myeloma activity of EDO-S101, a new bendamustine-derived molecule with added HDACi activity, through potent DNA damage induction and impairment of DNA repair
Source: J Hematol Oncol. 2017 Jun 20;10:127. doi: 10.1186/s13045-017-0495-y (PMC5477689; doi:10.1186/s13045-017-0495-y)
Supplement: Supplementary file 2 — Supplemental material and methods. (DOCX 127 kb) [file 13045_2017_495_MOESM2_ESM.docx]

**MM cell lines, patient samples and cultures.** Bone marrow (BM) samples from patients with MM were obtained after the approval of the Complejo Asistencial Universitario of Salamanca Review Board and after having obtained informed consent from participating subjects. Cells were cultured as previously described.^1^ Details of other tested compounds have been specified elsewhere.^2^

**Cell cycle and apoptosis assays.** The half-maximal inhibitory concentration (IC_50_) of the drug was calculated using the SigmaPlot software. The cell-cycle profile and apoptosis induction were evaluated using commercial kits provided by Immunostep (Salamanca, Spain) as described elsewhere.^3^ The percentage of cells at each cycle phase was calculated on the alive cells, not considering sub-G0 (apoptotic) cells in the computation.

**Immunohistochemistry anti-bodies.** Anti-cleaved PARP, anti-phospho-histone H2AX (Ser139), anti-acetyl-histone H3 (Cell Signaling, Boston, MA, USA) and anti-Ki67 (Thermo Scientific, Fremont, CA, USA).

**Western blot.** The origin of the primary antibodies used in western blot analyses was as follows: anti-pH2AX, anti-phospho-CHK1 (Ser345), anti-phospho-CHK2 (Thr68), anti-phospho-BRCA1 (1524), anti-phospho-ATM (Ser 1981), anti-phospho-ATR (Ser 428), anti-p53, anti-ac-histone 3, anti-acetyl-histone 4, anti-Bcl-XL, anti-Mcl1, anti-Bcl2 and anti-AIF (Cell Signaling, Boston, MA, USA). Horseradish peroxidase linked-donkey (anti-rabbit), sheep (anti-mouse) or mouse (anti-goat) immunoglobulins were used as secondary antibodies at 1:5000 dilution (Santa Cruz Biotechnology, Santa Cruz, CA, USA).

**Homologous recombination (HR) functional assay**. In the JJN3HR and U266HR with a plasmid integrated into the genome cells, a unique double strand break DSB may be introduced by the rare-cutting endonuclease *I-SceI*; a functional GFP gene is then reconstituted by gene conversion, the predominant HR repair pathway in mammalian cells. To evaluate HR efficiency in these cells, they were pre-incubated with various concentrations of EDO-S101 for 24 h. Then, 1x10^6^ cells were co-transfected with 5 µg of an *I-SceI*-expressing plasmid and 0.5 **μ**g of pDsRed-N1 to normalize for transfection efficiency, and incubated again in the presence or absence of EDO-S101 for 30 hours. Live cells were selected by FSC/SSC gating, and GFP+ and DsRed+ cells were quantified by flow cytometry. HR efficiency was calculated as the ratio of GFP+ to DsRed+ cells.

**Animal models.** Mice were randomized to the control group (receiving a PBS vehicle solution with 15% HPBCD, 1.5% acetic acid and 1.25% NaHCO_3_) or the treatment groups when tumors became palpable in the case of small plasmacytomas, or when the median tumor volume reached 4,000 mm^3^ for large plasmacytomas. To calculate the dose of each drug, a maximum tolerated dose (MTD) experiment in CB17-SCID mice was previously performed. The doses of bendamustine and vorinostat used in the final experiment are the MTD when both drugs were used in combination. All protocols and experiments were approved by the Animal Ethics Committee of the University of Salamanca.

Two *de novo* Vk*MYC mice with a M-spike corresponding to gamma/alpha ratio of 0.65 and 0.43 were chosen. EDO-S101 was administered once/week for two weeks by intra-cardiac injection at 30 mg/kg in vehicle solution. For transplantation studies, 7-10 week old C57BL/6J wt mice were transplanted with ~1x10^6^ million splenocytes harvested from Vk12653 tumor bearing mice. Drug treatment was performed as indicated for the *de novo* mice and was initiated once their M-spike levels reached >10g/L, or their gamma/albumin fraction was >0.3 to mimic clinical setting. Details on serum protein electrophoresis (SPEP) analysis have already been described.^2^

**Reagents and immunochemicals.** Cell culture media, fetal bovine serum and penicillin-streptomycin were purchased from Invitrogen Corporation (Gaithersburg, MD, USA). Bortezomib was purchased from LC Laboratories (Woburn, MA, USA), lenalidomide and pomalidomide from Selleckchem (Houston, TX, USA) and dexamethasone, vorinostat and mirin from Sigma-Aldrich (St Louis, MO, USA).

1 Maiso P, Carvajal-Vergara X, Ocio EM, López-Pérez R, Mateo G, Gutiérrez N *et al.* The histone deacetylase inhibitor LBH589 is a potent antimyeloma agent that overcomes drug resistance. *Cancer Res* 2006; **66**: 5781–5789.

2 Chesi M, Robbiani DF, Sebag M, Chng WJ, Affer M, Tiedemann R *et al.* AID-dependent activation of a MYC transgene induces multiple myeloma in a conditional mouse model of post-germinal center malignancies. *Cancer Cell* 2008; **13**: 167–80.

3 Maiso P, Carvajal-Vergara X, Ocio EM, López-Pérez R, Mateo G, Gutiérrez N *et al.* The histone deacetylase inhibitor LBH589 is a potent antimyeloma agent that overcomes drug resistance. *Cancer Res* 2006; **66**: 5781–5789.
